# Enhanced photon management in silicon thin film solar cells with different front and back interface texture

**DOI:** 10.1038/srep29639

**Published:** 2016-08-02

**Authors:** Asman Tamang, Aswin Hongsingthong, Vladislav Jovanov, Porponth Sichanugrist, Bakhtiar A. Khan, Rahul Dewan, Makoto Konagai, Dietmar Knipp

**Affiliations:** 1 Research Center for Functional Materials and Nanomolecular Science, Electronic Devices and Nanophotonics Laboratory, Jacobs University Bremen, Campus Ring 1, 28759 Bremen, Germany; 2 Department of Physical Electronics, Tokyo Institute of Technology, 2-12-1 Ookayama, Meguro-ku, Tokyo, 152-8550, Japan; 3 Photovoltaics Research Center (PVREC), Tokyo Institute of Technology, 2-12-1 Ookayama, Meguro-ku, Tokyo 158-0082, Japan

## Abstract

Light trapping and photon management of silicon thin film solar cells can be improved by a separate optimization of the front and back contact textures. A separate optimization of the front and back contact textures is investigated by optical simulations taking realistic device geometries into consideration. The optical simulations are confirmed by experimentally realized 1 μm thick microcrystalline silicon solar cells. The different front and back contact textures lead to an enhancement of the short circuit current by 1.2 mA/cm^2^ resulting in a total short circuit current of 23.65 mA/cm^2^ and an energy conversion efficiency of 8.35%.

Light trapping and photon management in silicon thin film solar cells are in the focus of intensive research due to its potential in increasing the short circuit current and energy conversion efficiency, while minimizing the material usage^1^. Most light trapping schemes are based on texturing of the front (solar cells in superstrate configuration) or back contact (solar cells in substrate configuration) of the solar cell^2^,^3^,^4^,^5^,^6^,^7^,^8^,^9^,^10^,^11^,^12^,^13^. Several research groups have tried to derive optimal surface textures using experimental and simulation approaches^5^,^6^,^7^,^8^,^9^,^14^. However, in order to allow for large area and low cost fabrication most silicon thin film solar cells are realized on randomly textured substrates^1^. By preparing the solar cells on randomly^2^,^3^,^4^,^15^,^16^ or periodically^3^,^5^,^6^,^7^,^14^ textured substrates the optical path length of the incident light in the solar cell is elongated. Consequently, quantum efficiency (QE) and short circuit current of the solar cell are also increased. The highest short circuit currents are achieved if both the front and back contacts of the solar cells are textured^4^,^5^,^7^,^14^,^15^. However, optimization of randomly textured substrates is a complex process. Several approaches have been developed to characterize the light scattering properties of textured substrates. Approaches ranging from haze measurements (ratio of diffuse to total transmission) to measurements of the Angular Distribution Function (ADF) have been developed^17^,^18^,^19^. Other approaches use the roughness of the substrates as input parameter to model the light trapping properties of solar cells on textured substrates^2^,^20^. More advanced approaches are presented using the Fourier Transform of the textured interfaces^21^ or surface analysis tools based on image processing^8^,^9^.

A detailed analysis of the optical simulations and experimental results reveals that a further increase of the short circuit current can be achieved if the front and back contact texture are separately optimized. However, the analysis and realization of such structures is complex. Optical simulations of thin crystalline silicon solar cells show that the highest short circuit current is achieved if different front and back textures are used^22^. However, it remains unclear how such crystalline silicon solar cell can be fabricated. In the case of silicon thin film solar cells several experimental studies are carried out, but very few simulation studies are performed to provide insights into the optics and to derive design rules for the optimization of such solar cells. Most experimental studies that were performed use multiscale textured substrates^10^,^12^,^16^,^23^,^24^,^25^,^26^,^27^. The multiscale textures consist of a combination of small and large surface textures. The multiscale texture allows for a separate control of the front and back contact textures. However, it is commonly argued by most authors that the small features are responsible for scattering of shorter wavelengths, while the larger surface features are responsible for scattering longer wavelengths. At the same time, most authors do not consider the influence of the back contact textures. High short circuit current can only be achieved by a proper design of the back contact textures. Hence, scattering processes and plasmonic losses of the metal back reflector have to be considered. This manuscript presents the first combination of both experimental and simulation studies of light trapping in microcrystalline silicon solar cells with different front and back contact texture.

In this study, a procedure to separately optimize the front and/or back contact surfaces of a 1 μm thick microcrystalline silicon (μc-Si:H) thin film solar cells prepared in superstrate configuration is carried out using optical simulations. Afterwards, optimal dimensions of the front and back contact textures are determined and used to define a substrate that allows for separate optimization of front and back contact textures. A 3D morphological algorithm is used to calculate realistic interface morphologies of each layer of the solar cells. The simulation results are confirmed by experimental results of solar cells considering the optimal front and back contact surfaces.

## Device Structure and Optical Simulation Model

**Figure 1:**
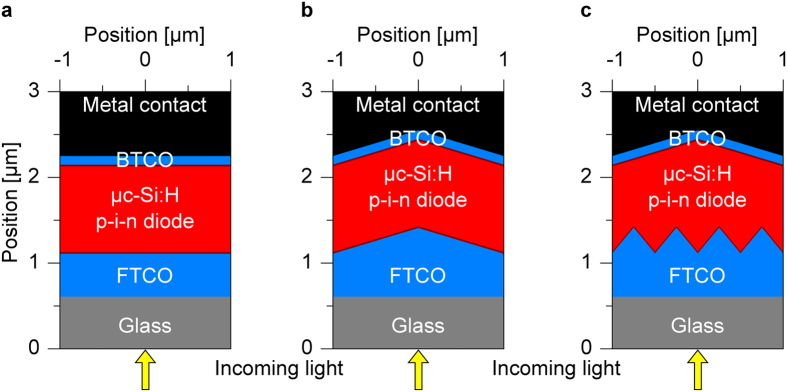
Schematic cross-sections of μc-Si: H thin film solar cells. The solar cells are prepared on (**a**) flat substrate, (**b**) identical front and back contact textures and (**c**) different front and back contact textures.

Cross-sections of the investigated μc-Si:H solar cell structures in superstrate configuration are shown in Fig. 1. The cross-sections of the solar cells represent a slice through the center of the unit cell of the simulated 3D solar cells. Figure 1a shows a reference solar cell on a flat substrate, while Fig. 1b shows a solar cell with identical front and back contact textures. Solar cells with identical interface textures represent the standard solar cells investigated in literature. Figure 1c exhibits a solar cell with different front and back contact textures. The front transparent conductive oxide (FTCO) layer of all solar cells consists of a 500 nm thick boron doped zinc oxide (ZnO:B). The μc-Si:H p-i-n diode consists of a 20 nm p-layer, 1 μm i-layer and 20 nm n-layer^5^,^7^,^10^,^14^,^15^. The back contact is defined by a 100 nm thick back TCO layer (back TCO, aluminum doped ZnO (ZnO:Al)) and a silver (Ag) back reflector. The optical constants of the materials of the solar cells are taken from literature^23^,^28^,^29^. The texture of the solar cells is approximated by a periodic arrangement of square shaped pyramids. The pyramids are placed in the direction of the substrate normal as shown in Fig. 1b and Supplementary Information Figs S1–S3. The period of the pyramid is varied from 0.1 to 5 μm, while the height of the texture is kept constant at 300 nm for all the simulations.

In this study, a Finite Difference Time Domain (FDTD) method is used to simulate the optical wave propagation within the solar cells in three dimensions^8^,^9^,^11^,^14^,^23^,^27^,^30^. The 3D FDTD simulations take the calculated 3D interface morphologies (see Supplementary Information Figs S1–S3) and film thickness as input parameters in order to model the 3D solar cell structures. Using the FDTD method, the electric field distributions are calculated for normal incidence light with an electric field amplitude of 1 V/m. Time average power loss profiles are calculated from the electric field distributions. Afterwards, the quantum efficiency (QE) is calculated as the ratio of the power absorbed in the i-layer with respect to the total power incident on the unit cell. It is assumed that all photo-generated charge carriers are collected. Electrical losses are not considered. Hence, the calculated quantum efficiency represents an upper limit. In order to take the charge transport properties into account the optical simulations have to be used as input parameter to simulate the optoelectronic properties in 1D^31^,^32^,^33^ or 3D^34^,^35^. However, by combining 3D optical with 3D electrical simulations the complexity of the simulations is significantly increased. In this study, we focus only on the optics of the solar cells. Therefore, it is assumed that the collection efficiency of the real solar cell is close to 100%. This assumption is valid because high quality microcrystalline silicon material is used in this study. Furthermore, the absorber layer of the solar cell is only 1 μm thick allowing for an excellent charge extraction. Finally, the short circuit current is calculated from the QE for 1.5 AM sun’s spectral irradiance. Details on the calculations of these parameters are given in literature^23^.

## Results

### Solar cells with textured front or back contacts

**Figure 2:**
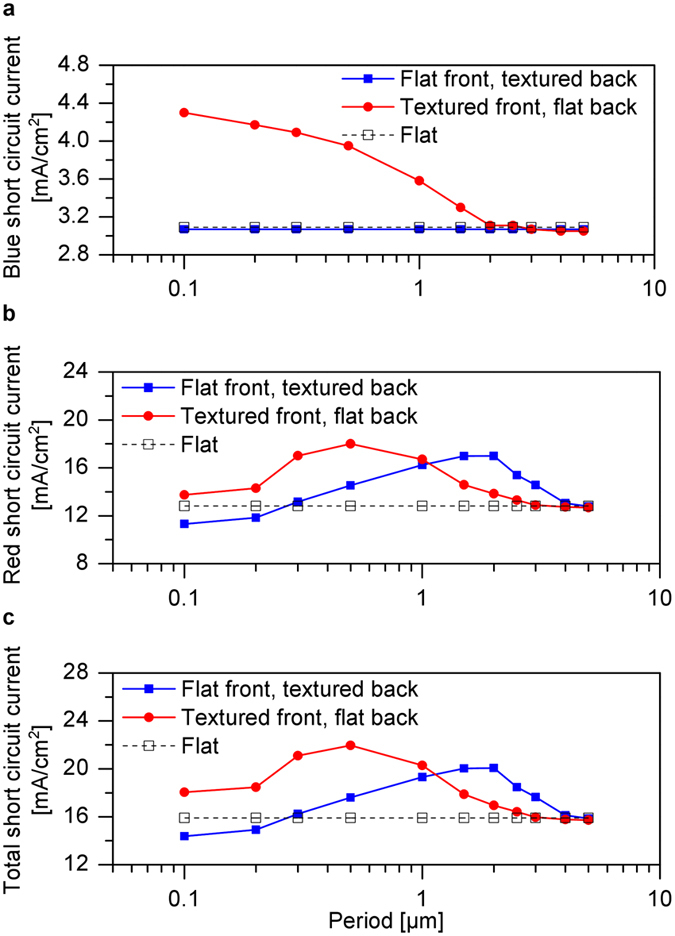
Short circuit currents for solar cells with flat contacts, textured front and flat back and textured back and flat front contacts. Calculated (**a**) blue, (**b**) red and (**c**) total short circuit currents.

In order to separate the influence of the front and back contact textures on the optics of the solar cell, solar cells with only one textured contact are investigated. In the first step, the optics of a μc-Si:H solar cells with textured front contact but flat back contact is investigated. In the second step, the back contact is textured, while the front contact is kept flat. The calculated short circuit currents of the solar cells are shown in Fig. 2. The short circuit current for shorter wavelengths in the blue spectral region (300–500 nm) and longer wavelengths in the red spectral region (500–1100 nm) are shown in Fig. 2a,b, respectively. The total short circuit current is shown in Fig. 2c. The textured front contact of a solar cell with flat back contact efficiently couples shorter wavelength light in the solar cell and scatters longer wavelengths light. The short circuit current for shorter wavelengths (300–500 nm) is maximized for a small period of 0.1 μm, while the short circuit current for longer wavelengths is maximized for a period of 0.5 μm. The total short circuit current is determined by the longer wavelengths light, so that the total short circuit current is maximized for a front contact texture with a period of 0.5 μm. For larger periods, the blue, red and total short circuit currents converge towards the reference flat solar cell (Fig. 1a). For a solar cell with flat front contact and textured back contact, the total short circuit current is maximized for the texture period of 1.5–2 μm (Fig. 2c). The short circuit current for shorter wavelengths is not affected by the back contact textures (Fig. 2a), because the shorter wavelength light gets absorbed close to the front contact of the solar cell. The longer wavelength light is efficiently scattered by the back contact resulting in high total short circuit current (Fig. 2b,c). It can be concluded from Fig. 2 that the short circuit current for shorter wavelengths is maximized if the front contact exhibits textures ranging from 0.1 μm to 0.5 μm. On the other hand, the short circuit current for longer wavelengths is maximized if the period of the back contact texture is in a range of 1.5–2 μm.

### Solar cells with different front and back surface textures

**Figure 3:**
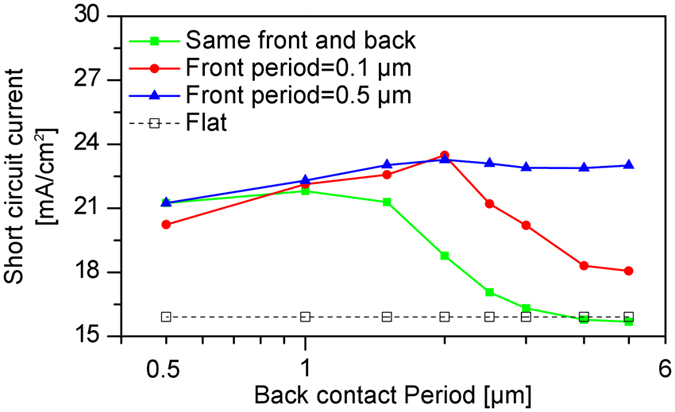
Short circuit currents of solar cells with same and different contacts as a function of back contact period. The solar cells are prepared on flat contacts, identical and different front and back contact textures.

In order to determine the optimal light trapping scheme, solar cells with different front and back contact textures are investigated. The period of the front texture is kept constant at 0.1 μm and 0.5 μm, while the period of the back contact texture is varied (Fig. 1c). The height of the surface texture is kept constant at 300 nm for all textured solar cells. Solar cells with flat interfaces (Fig. 1a) and identical front and back textures (Fig. 1b) are used as references. The simulation results for the solar cells with flat contacts, same or different textured contacts are summarized in Fig. 3. For the solar cells with different front and back contact textures, the short circuit current is maximized for a back texture period of 2 μm reaching a short circuit current of 23.5 mA/cm^2^. The short circuit current is increased by 1.7 mA/cm^2^ compared to an optimal reference solar cell with identical front and back contact textures. Only for the back texture period of 0.5–0.7 μm, the short circuit current of the reference solar cells is higher than that of solar cells with different front and back textures. However, for larger periods of the back contact textures (>0.9 μm), the short circuit current of the solar cells with different front and back contact textures is larger than that of the reference solar cells. For the solar cells with front texture period of 0.5 μm, the short circuit current remains constant for the back texture period larger than 1.5 μm. On the other hand, for the front texture period of 0.1 μm, the short circuit current drops for back texture periods larger than 2 μm. It can be concluded that solar cells with different front and back texture allow for a distinct increase of the short circuit current. In the following Sub-section, an approach is described on how to realize solar cells with different front and back contact.

### Solar cells with realistic interface textures

**Figure 4:**
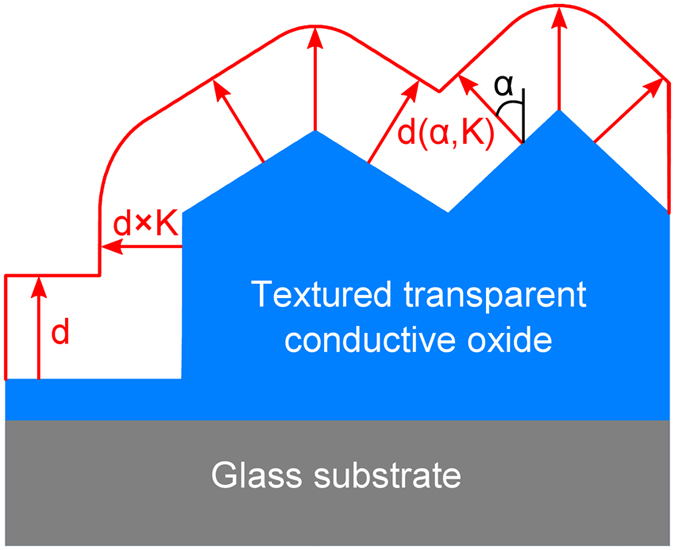
Modelling of the microcrystalline silicon film formation by 3D morphological algorithm. The local film thickness depends on the direction of the local surface normal and direction factor (K). The direction factor is 0.75.

The back contact morphology of a thin film solar cell in superstrate configuration is determined by the front contact morphology, the thickness of the silicon p-i-n diode and the deposition conditions of the silicon thin films. Hence, an independent control of the front and back contact morphologies is not possible using standard fabrication processes. The back contact morphology can only be controlled by properly designing the front contact morphology. In this study, a multiscale textured substrate is used to achieve different front and back contact textures. The multiscale textured solar cell consists of a combination of a large and small surface textures to act as a front TCO layer, where small surface textures are formed on top of the larger ones. By depositing a silicon film on such a surface texture, the back contact textures are determined mainly by the large front contact textures, while the small surface features are smoothened out. The formation of the silicon thin film on textured substrates is schematically illustrated in Fig. 4. In order to calculate the morphology of the silicon film, a 3D morphological algorithm is used. The morphological algorithm is based on the assumption that the growth direction of the silicon film can be described by the local surface normal. The input parameters of the morphological algorithm are the substrate morphology and nominal thickness of the film (d). To obtain the morphology of the deposited film, the morphological algorithm determines the local surface normal for each position on the substrate. In the next step, the film thickness (d(α,K)) is calculated for each substrate point assuming that it depends on the angle (α) between the local surface normal and glass substrate normal as shown in Fig. 4. This dependence is described by a direction factor, K. The thickness is equal to the nominal value (d), if the local surface normal is parallel to the substrate normal (α = 0°). On the other hand, if the local surface normal is orthogonal to the glass substrate normal (α = 90°), the thickness is reduced to d×K. More details on the film formation and the morphological algorithm can be found in literature^36^,^37^. The model is derived by comparing measured and simulated surfaces morphologies of microcrystalline silicon films prepared on randomly textured substrates^37^. The direction factor is determined to be K = 0.75 for microcrystalline silicon films and K = 1 for amorphous silicon films^37^.

**Figure 5:**
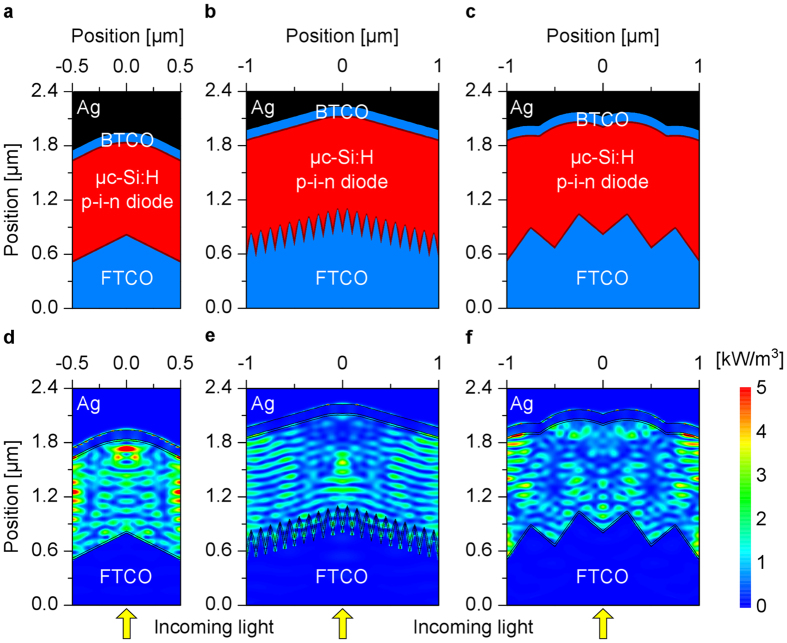
Cross-sections and power loss profiles of solar cells. (**a**) Single, multiscale textured with small textures period of (**b**) 0.1 and (**c**) 0.5 μm. Respective power loss profiles (**d**–**f**) for 700 nm wavelength.

Cross-sections of solar cells with single and multiscale textured substrates are shown in Fig. 5a–c. The interface morphologies of the consecutive layers of the solar cells are calculated using the morphological algorithm, which uses the surface morphology of the textured front TCO layer and the film thicknesses as input parameters. Figure 5d–f exhibits the corresponding calculated power loss profiles of the solar cell structures for an incident wavelength of 700 nm. The power loss profiles show a slice through the center of the 3D power loss profiles. The simulations show that longer wavelengths light (700 nm) reaches the metal back contact, where some portion of light is absorbed, while most of the light is reflected (Fig. 5d–f). Furthermore, the absorption of light in the silicon film is increased due to scattering/diffraction of the incident light by the textured contacts (Fig. 5d–f). The absorption of light for each layer of the solar cells is shown in Supplementary Information Fig. 4S and Table S1. The back contact losses are the sum of absorptions in n-layer of p-i-n diode, back TCO layer and metal back contact. Figure 5a exhibits the cross-section of the reference single textured solar cell, where the μc-Si:H film is formed on top of a single front texture. A period of 1 μm is selected for the reference solar cell, because the highest short circuit current is observed for this period (Fig. 3). Figure 5b,c exhibit the cross-sections of the solar cells on multiscale textured substrates. The multiscale textured solar cells consist of small textures with a period of 0.1 μm (Fig. 5b) and 0.5 μm (Fig. 5c) in combination with a big surface texture with a period of 2 μm. The small textures are placed on the big surface texture in the direction of the glass substrate normal as shown in Fig. 5b,c and Supplementary Information Figs S2 and S3. The height of all the textures is assumed to be 300 nm. The silicon film growth leads to a smoothening of the surface textures, so that the small surface textures are smoothed out. The back contact morphology is determined by the large front surface texture.

**Figure 6:**
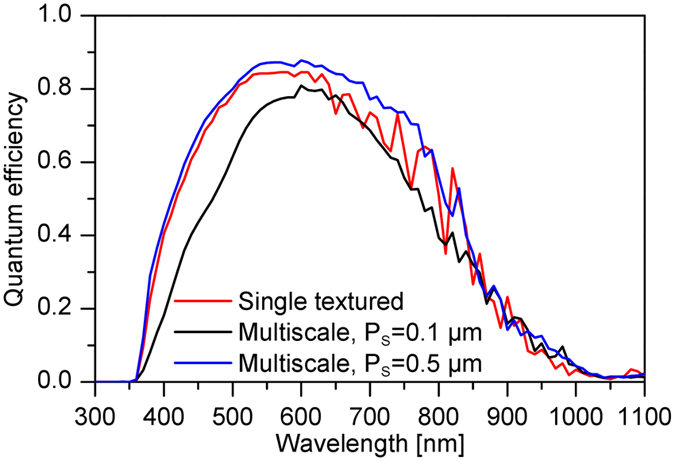
Quantum efficiencies of μc-Si:H solar cells. The solar cells are prepared on single textured substrate and multiscale textured substrate with small textures of (P_S_) of 0.1 μm and 0.5 μm.

The quantum efficiencies of the simulated solar cells are depicted in Fig. 6. The multiscale textured solar cell with 0.5 μm period of small textures (Fig. 5c) shows a significant improvement of the QE compared to the single textured solar cell (Fig. 5a) and multiscale textured solar cell with 0.1 μm period of small textures (Fig. 5b). An improved QE is observed for almost the entire wavelength range resulting in a short circuit current of 23 mA/cm^2^ (Fig. 6). The short circuit currents of 21.6 mA/cm^2^ and 19 mA/cm^2^ are obtained for the single and multiscale (0.1 μm period of small textures) textured solar cells, respectively. The lowest short circuit current is observed for the multiscale textured solar cell with 0.1 μm period of small textures. The increased effective (averaged) thickness of the p-layer leads to an increased absorption in the p-layer. The effective thickness is defined as the averaged local thickness of the thin film on the substrate^23^,^27^,^36^. For the multiscale textured solar cell with 0.1 μm period of small textures, the effective thickness of the p-layer is calculated to be 80 nm assuming a nominal thickness of 20 nm. On the other hand, the effective thickness of the p-layer of the single and multiscale (0.5 μm period of small textures) textured solar cells is 25 nm for the same nominal thickness. The loss in the p-layer is shown in Supplementary Information Fig. S4 and quantified in Table S1. As a result, a drop of the QE for shorter wavelengths is observed (Fig. 6). Furthermore, the back contact texture is determined by the big front surface texture (2 μm), while the small front surface textures does not have an influence on the back contact morphology. Consequently, the back contact losses in the multiscale (0.1 μm period of small textures) textured solar cell are smaller compared to the reference solar cell and multiscale textured solar cell with 0.5 μm period of small textures (Supplementary Information Fig. S4 and Table S1). It can be concluded that the period of the small textures of the multiscale textured solar cell should be larger than 0.1 μm to reduce the optical losses in the p-layer and increase the total short circuit current. A single textured solar cell with front texture period of 2 μm (not shown in Fig. 6) leads to a short circuit current of 18.6 mA/cm^2^. Therefore, the multiscale textured solar cell combining the small textures with period of 0.5 μm and a large texture with period of 2 μm provides the best results. A gain of short circuit current of 1.4 mA/cm^2^ is obtained for the multiscale textured solar cell compared to the optimized single textured solar cell.

### Experimental realization of multiscale textured solar cells

**Figure 7:**
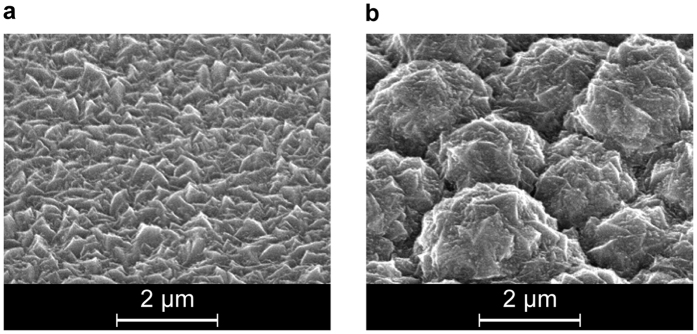
Scanning Electron Microscope images of boron doped ZnO film prepared on (**a**) flat and (**b**) etched glass substrate.

In the following, the fabrication of randomly textured substrates for single and multiscale textured solar cells is described. The single and multiscale textured front TCO layer is prepared on flat and textured glass substrate, respectively. In this part of the study, the solar cell on the flat glass substrate is used as reference. The glass substrates of the multiscale textured solar cell are textured by using a reactive ion etching process with carbon tetrafluoride (CF_4_) as an etchant gas. The plasma treatment has been carried out at a gas pressure of 13 Pa and power density of 1.5 W/cm^2^. After the plasma treatment, the glass substrate exhibits a root mean square (rms) roughness of 360 nm. The front TCO layer is prepared by depositing ZnO:B films onto the etched glass substrates by using metal-organic chemical vapor deposition (MOCVD) technique using water as an oxidant for diethylzinc. Scanning Electron Microscope (SEM) images of single and multiscale textured substrate are shown in Fig. 7. Details on the fabrication of the single and multiscale textured substrate are given in literature^12^,^23^,^27^. The solar cell on the single textured substrate is characterized by small features with an average size of 300 nm, while the multiscale substrate is characterized by large features with an average size of 2 μm. Same as for the substrate with the single texture, the average size of the small features which are formed on top of the larger feature is 300 nm. The μc-Si:H p-i-n diode is fabricated on top of the textured front TCO layer by plasma enhanced chemical vapor deposition (PECVD)^13^,^38^,^39^. Wide band gap silicon oxide (Si_1−x_O_x_) layers are used as p-layer of the p-i-n diode to minimize the optical loss in the p-layer^7^,^14^,^23^,^27^,^40^,^41^. The p-layer, i-layer and n-layer of the p-i-n silicon diode exhibit a thickness of 20 nm, 1 μm and 20 nm, respectively. Afterwards, a 100 nm thick ZnO layer is prepared on top of the silicon film to reduce parasitic losses in the metal back reflector^3^,^11^,^40^,^42^,^43^. Finally, a Ag back reflector is deposited.

**Figure 8:**
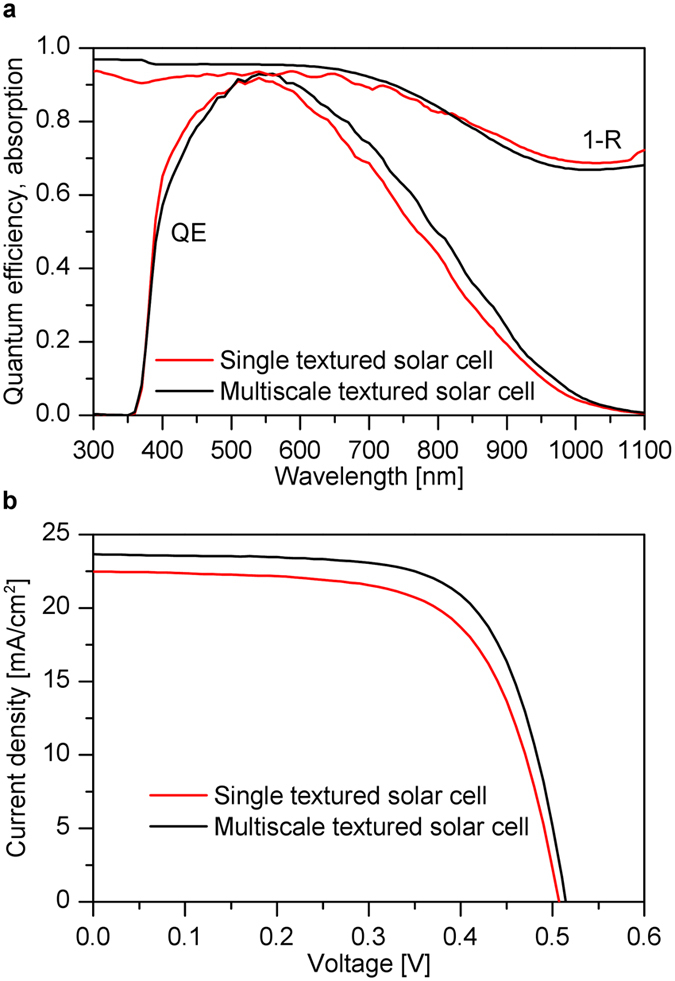
Measured optical and electrical properties of the single and multiscale textured μc-Si:H thin film solar cells. Measured (**a**) quantum efficiencies (QE) and absorptions (1-R) and (**b**) current/voltage characteristic of the solar cells.

The measured quantum efficiency and total absorption (1-R) of the single and multiscale textured solar cells are shown in Fig. 8a. The QE is measured under short circuit current conditions. The multiscale textured solar cell exhibits a small decrease of the QE for shorter wavelengths (380–520 nm) and distinct increase for longer wavelengths (>600 nm) compared to single textured solar cell. A comparison with the simulation results illustrated in Fig. 6 exhibits a small difference for shorter wavelengths. The difference might be caused by the fact that the effective thickness of the p-layer for the experimentally realized multiscale textured solar cell is larger. For longer wavelengths a comparison of the measured (Fig. 8a) and simulated (Fig. 6) quantum efficiencies exhibits a good agreement.

The current/voltage characteristics of the single and multiscale textured solar cells under AM 1.5 illumination is shown in Fig. 8b. The single textured solar cell exhibits a short circuit current of 22.52 mA/cm^2^, fill factor of 66% and an open circuit voltage of 505 mV yielding an energy conversion efficiency of 7.5%. The multiscale textured solar cell exhibits a short circuit current of 23.65 mA/cm^2^, fill factor of 69%, and an open circuit voltage of 515 mV resulting in the conversion efficiency of 8.35%. Thus, a gain in the short circuit current of 1.2 mA/cm^2^ is achieved for the multiscale textured solar cell compared to the single textured solar cell. Further gain in the open circuit voltage and fill factor results to increased conversion efficiency in the multiscale textured solar cell.

## Discussion

The back contact morphology depends on the front contact morphology, the solar cell thickness and the deposition conditions of the silicon thin film. Hence, different aspects have to be considered when developing strategies to control the back contact morphology. The use of multiscale textured substrates provides a promising approach to control the front and back contact morphologies. Different authors have presented their studies on multiscale textured substrates^10^,^12^,^16^,^23^,^24^,^25^,^26^,^27^. However, a detailed understanding of the optical wave propagation in multiscale textured solar cells is still missing. Most authors argue that shorter wavelength light is diffracted by the small surface features, while the longer wavelength light is diffracted by the larger surface features. However, the situation is more complex. Table 1 provides a relationship between the dimensions of the surface texture and the thickness of the solar cell. The period or diameter of the large feature (P_L_), the period of the small features (P_S_), and the thickness of the solar cell (t) are used to distinguish different cases.

The short circuit current of a solar cell on a single texture is maximized if the period (P) is approximately equal to the thickness of the solar cell^7^,^14^. For smaller periods, the longer wavelength light is not efficiently diffracted. For larger periods, the diffraction angle gets smaller, so that only a small increase of the absorption of the light is observed. However, for multiscale textured solar cell, the situation is more complex.

In the first case P_S_≈t and P_L_ ≫ t: If P_S_ is approximately equal to the thickness of the solar cell, the light is efficiently scattered. P_L_ is significantly larger than the thickness of the solar cell, so that the light is diffracted in small angles. However, the short circuit current is increased due to the refraction of the incident light at the glass/ZnO or ZnO/silicon interface. This is confirmed by optical simulations shown in Fig. 3 (front texture period of 0.5 μm, back texture period of 5 μm). Furthermore, this is partly confirmed by the experimental results presented in Sub-section 3.D.

In the second case P_L_ ≫ t and P_S_ ≪ t: If P_S_ is distinctly smaller than the thickness of the solar cell, the features are too small to efficiently scatter the incident light. The small features allow for an improved incoupling of mainly shorter wavelengths. However, the gain due to improved light incoupling is small compared to the gain achieved by the efficient scattering of longer wavelengths due to larger surface features. Hence, the surface feature does not represent an optimal surface texture. This is supported by the calculated short circuit current shown in Fig. 3 (front texture period of 0.1 μm, back texture period of 5 μm).

In the third case P_L_≈t and P_S_≫t: If P_L_ is approximately equal to the thickness of the solar cell, the light is efficiently diffracted. P_S_ is significantly smaller than the thickness of the solar cell, so that an improved incoupling of the light is observed as shown in Fig. 3 (front texture period of 0.1 μm, back texture period of 2 μm).

However, besides the optics, further aspects have to be considered. The growth of the silicon thin film on textured substrates leads to an increased effective thickness of the silicon thin film solar cell. The effective thickness is larger than the nominal thickness, when the thin film is deposited on the textured substrates. The effective thickness increases with increasing roughness of the substrates. However, the increased thickness is observed for all layers of the solar cell. Hence, the QE and short circuit current of solar cells with P_L_≈t and P_S_ ≪ t are limited by absorption losses. The increased effective thickness of the p-layer leads to optical losses for shorter wavelengths shown in Fig. 6, Supplementary Information Fig. S4 and Table S1 (Multiscale, P_S_ = 0.1 μm). Hence, a high short circuit current can not be achieved.

Furthermore, the formation of the films is closely related to the microstructure of the μc-Si:H films^7^,^30^,^44^,^45^,^46^,^47^. Regions of reduced structural order are potentially formed in the μc-Si:H film. The regions of reduced structural order are commonly called “cracks” which negatively affect fill factor and open circuit voltage of the solar cells^30^,^44^,^45^,^46^,^47^. Secondary ion mass spectrometry (SIMS) measurements reveal that the oxygen concentration is increased in the regions of reduced structural order resulting in an increased concentration of recombination centers^30^,^47^. The concentration of cracks depends on the surface morphology, the thickness of the silicon films, and the deposition conditions^7^,^14^,^30^,^44^,^45^,^46^,^47^. Therefore, a careful control of the surface morphology is required to avoid the formation of cracks. However, the multiscale textured solar cell might be sensitive to the formation of cracks.

Finally, the metal back reflector of a solar cell has a distinct influence on the optics of the solar cell. The growth of the μc-Si:H film leads to the formation of nano features. The propagation of the nano features all the way to the metal reflector can cause significant plasmonic optical losses^11^,^48^,^49^. Furthermore, the shape of the metal back reflector has a distinct influence on optical losses of the back reflector. In order to minimize optical losses, the formation of nano features has to be suppressed.

In a previous study, we have investigated the influence of multiscale texture on the optics of amorphous silicon solar cells^23^,^27^. The optics of amorphous silicon solar cells on multiscale textured substrates is fundamentally different from microcrystalline silicon solar cells on multiscale textured substrate. Nevertheless, preparing amorphous silicon solar cells on multiscale textured substrates lead to a gain of the short circuit current. This effect is not caused by scattering since the large surface textures are too large to efficiently scatter longer wavelengths. The large surface textures refract the incident light in larger angles. The gain in the short circuit current is mainly caused by a distinctly increased effective thickness of the absorber layer (amorphous silicon) of the solar cell. For the absorber layer of a microcrystalline silicon solar cell on a multiscale textured substrate the effect is rather small, because the absorber layer thickness of microcrystalline silicon solar cell is distinctly larger than the thickness of an amorphous silicon solar cell. Furthermore, the larger surface texture is too large to efficiently scatter visible light, while the same sized surface textures allows for scattering of infrared light in a microcrystalline silicon solar cell. Nevertheless, a gain in both the short circuit current from 15.5 mA/cm^2^ to 16.8 mA/cm^2^ and the energy conversion efficiency from 9.5% to 10.7% is observed when the amorphous silicon solar cells are prepared on multiscale textured substrates instead of single textured substrates^23^,^27^.

The multiscale textured substrate holds the potential to significantly improve the performance of silicon thin film solar cells. Multiscale textured substrates are ideal substrates for the realization of multi-junction solar cells. In the first step, an amorphous silicon solar cell is prepared on the multiscale textured substrate. The small features allow for the light trapping of shorter wavelength in the top diode. Furthermore, the formation of cracks is suppressed. The large surface features propagate through the layer stack of the amorphous silicon p-i-n top diode. In the second step, the microcrystalline silicon bottom diode is prepared. The large surface feature allows for an efficient refraction of the light in the bottom cell of the tandem solar cell. Experimentally realized solar cell on multiscale textured substrates exhibits an energy conversion efficiency of 13.2%^25^. By minimizing the optical losses in the solar cell energy conversion efficiencies of up to 14.8% have been demonstrated^26^.

## Summary

The light trapping properties of silicon thin film solar cells can be improved by separately optimizing the front and the back contact textures. The optics in the solar cells is studied experimentally and numerically. The front texture of the solar cell efficiently couples light in the solar cell and scatters shorter wavelength light, while the texture of the back contact scatters longer wavelengths light. Optimal surface textures are determined by taking realistic interface morphology into account. Multiscale textured substrates are used to separately control the morphology of the front and the back contact. The interface morphology of the solar cells is calculated by a 3D morphological algorithm. A multiscale textured solar cell with small textures of period of 0.5 μm and a micro-texture of period of 2.0 μm exhibits the highest short circuit current. Finally, 1 μm thick multiscale textured μc-Si:H solar cells are fabricated. The large surface textures are realized by etching the glass substrate. The small textures on the front TCO layer are determined by the LPCVD deposition of ZnO. The multiscale textured solar cell leads to a gain of the short circuit current of 1.2 mA/cm^2^ from 22.52 mA/cm^2^ to 23.65 mA/cm^2^ compared to single textured solar cell resulting in a conversion efficiency of 8.35%.

## Additional Information

**How to cite this article**: Tamang, A. *et al*. Enhanced photon management in silicon thin film solar cells with different front and back interface texture. *Sci. Rep.* **6**, 29639; doi: 10.1038/srep29639 (2016).

## Supplementary Material

Supplementary Information

## Figures and Tables

**Table 1:** Guideline for the optimization of the surface texture of single and multiscale textured silicon thin film solar cells.

**Single textured solar cell**			**Multiscale textured solar cell**		
**Feature size**	**Effect**	**Short circuit current**	**Feature size**	**Effect**	**Short circuit current**
P < t	longer wavelengths are not diffracted	low	P_L_ ≫ tP_S_≈t	Small features diffract light, low absorption losses	high
P≈t	Diffraction	high	P_L_ ≫ tP_S_ ≪ t	Small features improve light incoupling, high absorption losses	low
P > t	Diffracted in small angles	low	P_L_≈tP_S_ ≪ t	Small features improve light incoupling, high absorption losses	low
