# Supplementary material for: Enhanced photon management in silicon thin film solar cells with different front and back interface texture
Source: Sci Rep. 2016 Aug 2;6:29639. doi: 10.1038/srep29639 (PMC4969606; doi:10.1038/srep29639)
Supplement: Supplementary Information [file srep29639-s1.pdf]

Enhanced photon management in silicon thin film solar cells with different front and back interface texture

Asman Tamang[1], Aswin Hongsingthong[2], Vladislav Jovanov[1], Porponth Sichanugrist[2,4], Bakhtiar A. Khan[1], Rahul Dewan[1], Makoto Konagai[2,3,4,5] & Dietmar Knipp[1,6,*]

[1]Research Center for Functional Materials and Nanomolecular Science, Electronic Devices and Nanophotonics Laboratory, Jacobs University Bremen, Campus Ring 1, 28759 Bremen, Germany

[2]Department of Physical Electronics, Tokyo Institute of Technology, 2-12-1 Ookayama, Meguro-ku, Tokyo, 152-8550, Japan

[3]Photovoltaics Research Center (PVREC), Tokyo Institute of Technology, 2-12-1 Ookayama, Meguro-ku, Tokyo 158-0082, Japan

[4]now with MEXT/FUTURE-PV Innovation Research, JST, Fukushima 963-0215, Japan

[5]now with Advanced Research Lab., Tokyo City University, 8-5-1, Todoroki, Setagaya-ku, Tokyo 158-0082, Japan

[6]currently with Geballe Laboratory for Advanced Materials, Department of Materials Science and Engineering, Stanford University, Stanford, CA 94305, USA

*.) Corresponding author: dietmarknipp@gmail.com (D. Knipp)

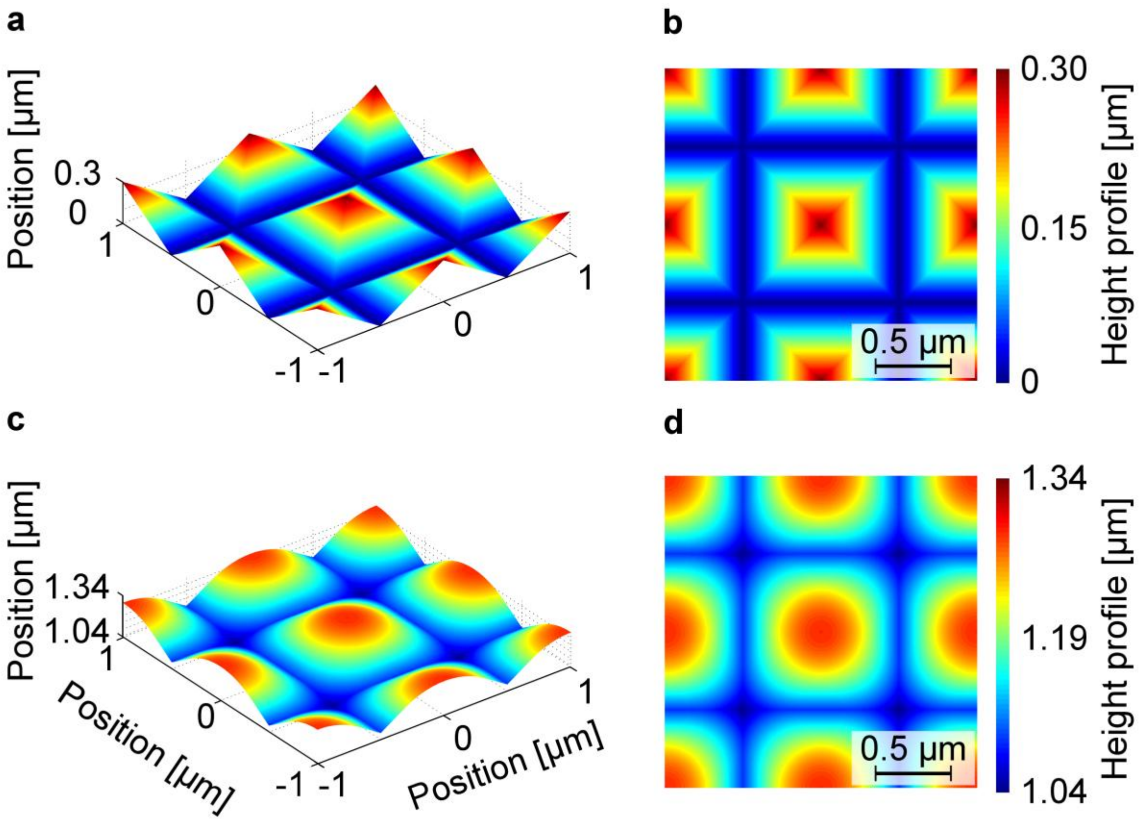

Supplementary Figure S1. Computationally generated surface morphologies for single textured solar cell. (a) Side and (b) top views of front TCO layer. (c) Side and (d) top views of silicon film.

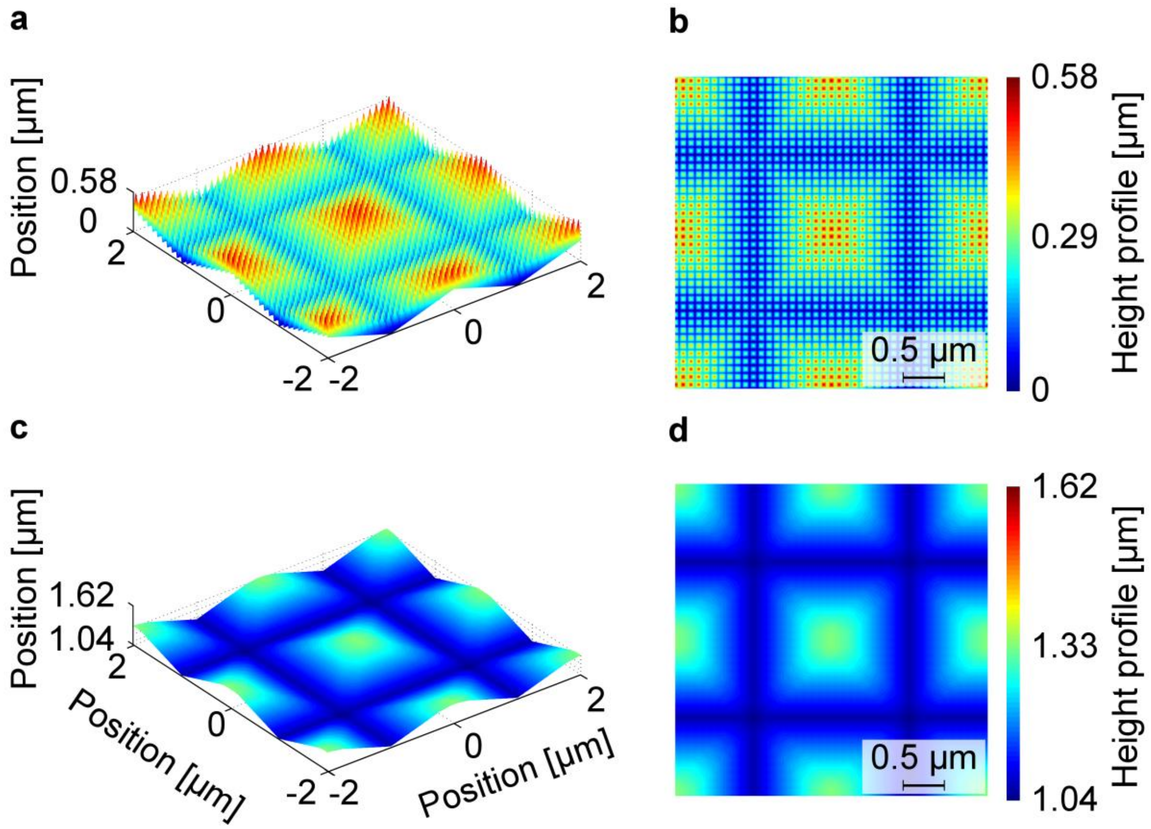

Supplementary Figure S2. Computationally generated surface morphologies for multiscale textured solar cell with small textures period of 0.1 μm. (a) Side and (b) top views of front TCO layer. (c) Side and (d) top views of silicon film.

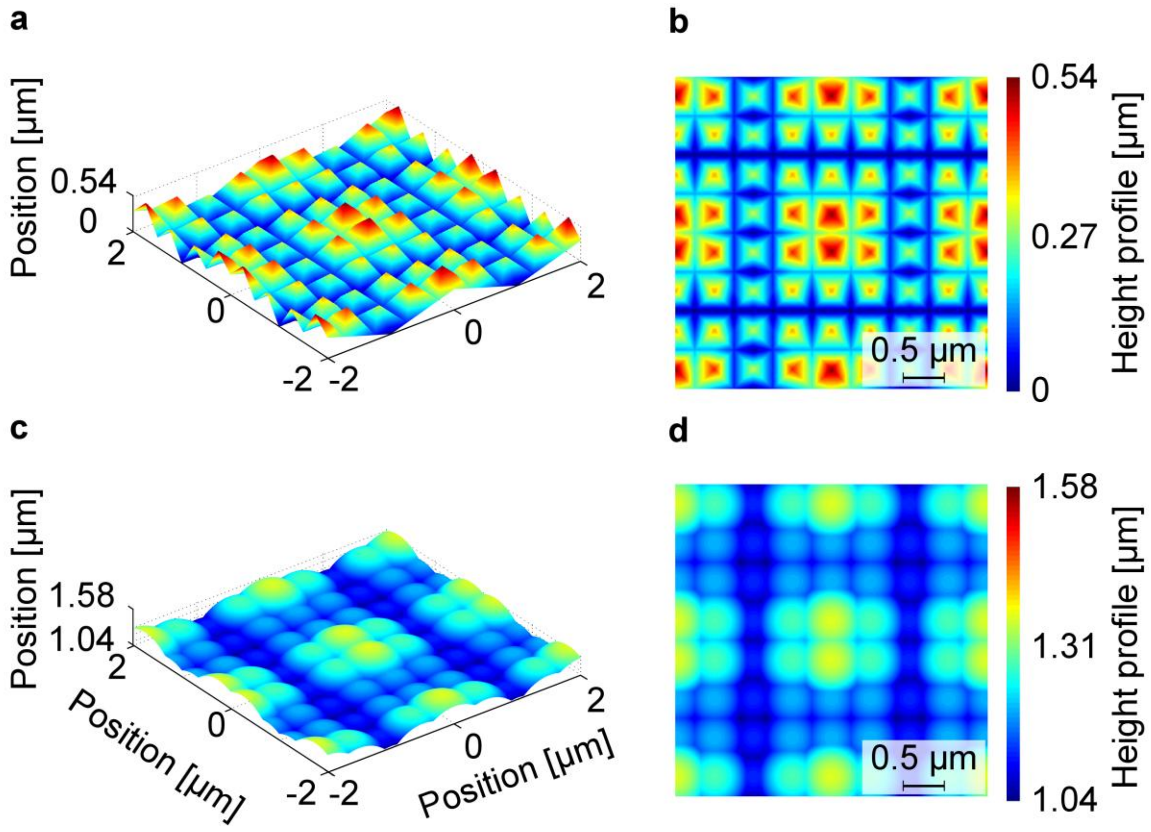

Supplementary Figure S3. Computationally generated surface morphologies for multiscale textured solar cell with small textures period of 0.5 μm. (a) Side and (b) top views of front TCO layer. (c) Side and (d) top views of silicon film.

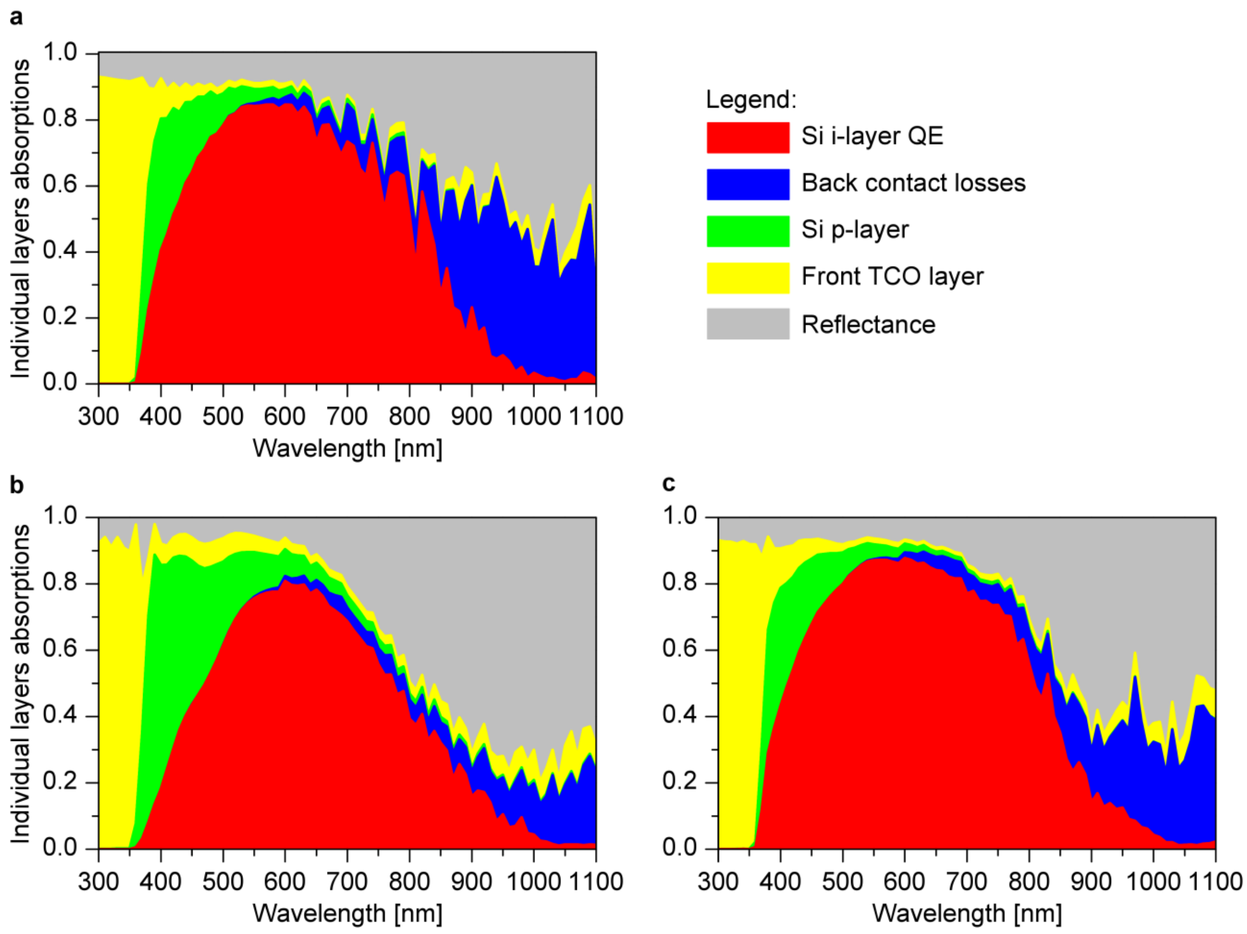

Supplementary Figure S4. Individual layers absorptions of μc-Si:H solar cells.

Simulated results are (a) single textured solar cell, multiscale textured solar cell with period of small textures of (b) 0.1 μm and (c) 0.5 μm.

Supplementary Table S1. Calculated currents for individual layers absorption of μc-Si:H solar cells with single textured solar cell and multiscale textured interfaces.

	**Texture of solar cell interfaces**		
	**Single texture P=1 μm**	**Multiscale texture, P_L=2 μm, P_S=0.1 μm**	**Multiscale texture, P_L=2 μm, P_S=0.1 μm**
**Short circuit current [mA/cm^2]**	21.6	19.0	23.0
**Back contact losses [mA/cm^2]**	6.3	2.6	4.5
**Silicon p-layer [mA/cm^2]**	1.9	4.3	1.8
**Front TCO layer [mA/cm^2]**	2.3	2.9	2.4
**Reflectance losses [mA/cm^2]**	10.5	14.0	11.1
